# Global patterns of crop yield stability under additional nutrient and water inputs

**DOI:** 10.1371/journal.pone.0198748

**Published:** 2018-06-27

**Authors:** Christoph Müller, Joshua Elliott, Thomas A. M. Pugh, Alex C. Ruane, Philippe Ciais, Juraj Balkovic, Delphine Deryng, Christian Folberth, R. Cesar Izaurralde, Curtis D. Jones, Nikolay Khabarov, Peter Lawrence, Wenfeng Liu, Ashwan D. Reddy, Erwin Schmid, Xuhui Wang

**Affiliations:** 1 Potsdam Institute for Climate Impact Research, Potsdam, Germany; 2 University of Chicago and ANL Computation Institute, Chicago, Illinois, United States of America; 3 Columbia University Center for Climate Systems Research, New York, New York, United States of America; 4 School of Geography, Earth & Environmental Science and Birmingham Institute of Forest Research, University of Birmingham, Birmingham, United Kingdom; 5 IMK-IFU, Karlsruhe Institute of Technology, Garmisch-Partenkirchen, Germany; 6 National Aeronautics and Space Administration Goddard Institute for Space Studies, New York, New York, United States of America; 7 Laboratoire des Sciences du Climat et de l’Environnement, Gif-sur-Yvette, France; 8 Ecosystem Services and Management Program, International Institute for Applied Systems Analysis, Laxenburg, Austria; 9 Department of Soil Science, Faculty of Natural Sciences, Comenius University in Bratislava, Bratislava, Slovak Republic; 10 Climate Analytics, Berlin, Germany; 11 University of Maryland, Department of Geographical Sciences, College Park, Maryland, United States of America; 12 Texas A&M University, Texas AgriLife Research and Extension, Temple, Texas, United States of America; 13 National Center for Atmospheric Research, Earth System Laboratory, Boulder, Colorado, United States of America; 14 Eawag, Swiss Federal Institute of Aquatic Science and Technology, Duebendorf, Switzerland; 15 Institute for Sustainable Economic Development, University of Natural Resources and Life Sciences, Vienna, Austria; 16 Sino-French Institute of Earth System Sciences, Peking University, Beijing, China; Instituto Agricultura Sostenible, SPAIN

## Abstract

Agricultural production must increase to feed a growing and wealthier population, as well as to satisfy increasing demands for biomaterials and biomass-based energy. At the same time, deforestation and land-use change need to be minimized in order to preserve biodiversity and maintain carbon stores in vegetation and soils. Consequently, agricultural land use needs to be intensified in order to increase food production per unit area of land. Here we use simulations of AgMIP’s Global Gridded Crop Model Intercomparison (GGCMI) phase 1 to assess implications of input-driven intensification (water, nutrients) on crop yield and yield stability, which is an important aspect in food security. We find region- and crop-specific responses for the simulated period 1980–2009 with broadly increasing yield variability under additional nitrogen inputs and stabilizing yields under additional water inputs (irrigation), reflecting current patterns of water and nutrient limitation. The different models of the GGCMI ensemble show similar response patterns, but model differences warrant further research on management assumptions, such as variety selection and soil management, and inputs as well as on model implementation of different soil and plant processes, such as on heat stress, and parameters. Higher variability in crop productivity under higher fertilizer input will require adequate buffer mechanisms in trade and distribution/storage networks to avoid food price volatility.

## Introduction

Agricultural production of feed and food commodities is subject to variations in weather, management and other environmental conditions [1]. Large perturbations in agricultural production by weather phenomena such as the 2012 drought in the US, 2010 flooding in Pakistan, 2003 heat wave in Europe, can lead to local and global price spikes of food commodities that can endanger food security, especially for low-income population shares and subsistence framers [2, 3]. Trade and related policies such as food storage strategies can help to alleviate the negative impact of variations in agricultural crop production on food supply [4], especially in nations with the resources to access and implement these. Nonetheless, even non-subsistence farmers also strive to avoid negative impacts of weather fluctuations on their production and subsequently their income. The susceptibility to weather-driven yield variations is affected not only by the occurrence of adverse weather conditions but also by the nature of the agricultural management in place.

In the context of on-going increases in demand for agricultural products resulting from both increases in population and shifts in dietary preferences [5], there has been considerable interest in potential production gains resulting from intensification of agriculture, i.e. increasing management inputs in order to increase yields [6]. Such intensification is well known to have substantial environmental consequences that extend well beyond the agricultural systems themselves, for instance the eutrophication of surface waters through leaching of nitrogen fertilizer (e.g., [7]) or the acidification of forests through nitrogen deposition [8]. It is also very resource intensive, often requiring a high energy input or irrigation water that exceeds local availability [9]. This has led to a widespread debate as to the relative merits of intensification versus extensification for mean yields and land use change or other environmental consequences [10, 11]. Yet the influence of intensification on interannual yield variability has been much less explored. Maximizing mean yield through intensification may come at the cost of yield reliability [12], with implications for both food and economic security.

Here we explore the role of additional inputs of nutrients and water on the stability of crop yield production. Nutrient supply in form of mineral or organic inputs and water supply are the most limiting resources in agricultural production and are used extensively for agricultural production [13]. While the term “yield stability” can describe the full genome-environment interaction (G x E) especially in the context of breeding [14], we here use the term “yield stability” only with respect to variations in time, i.e. high yield stability implies low inter-annual yield variability. For our analysis we use results for the entire simulation data set available for the period 1980 to 2009 from a large crop model ensemble that was run for current management conditions, as well as for scenarios in which nutrients and/or water are assumed to be available in unlimited supply. This data set is part of the Global Gridded Crop Model Intercomparison (GGCMI) Project [15] within the Agricultural Model Intercomparison and Improvement Project (AgMIP) [16] and the InterSectoral Impact Model Intercomparison Project (ISIMIP) [17]. The objective of these projects is to quantify uncertainty in model projections, understand and reduce sources of model disagreement by conducting protocol-based model ensemble experiments with structured harmonization levels across models. The data set used here allows for testing the role of additional inputs on crop yield stability of the major four crops maize (*Zea mays L*.), wheat (*Triticum aestivum L*.), rice (*Oryza sativa L*.) and soybeans (*Glycine max L*.). Simulation data for other crops, even though also important for human nutrition, are not available in this data set. The models and modeling frameworks contributing to this data set have been evaluated at global, national and grid-cell level and were found to perform reasonably well with respect to spatial and temporal dynamics, but with substantial differences between individual GGCMs [18]. While most GGCMs (8 of 10) are able to reproduce a statistically significant fraction of observed yield variability for maize and wheat, with the model setup used here as the baseline, only 2 models do so for rice and 5 out of 9 do so for soybean (Table A in S1 File). Generally, GGCMs tend to better reproduce observed yield variability in intensely managed countries, where it can be assumed that the observed yield variability is mainly weather driven. Global analyses suggest that about one third of observed yield variability is caused by weather variability, with substantial regional variation of the weather-induced variability [19]. As the evaluation of these models is very complex, we refer readers to Müller, Elliott [18] for further details on model performance. Through the use of a large ensemble of crop models however, we are able to effectively characterize yield variability [20] thereby addressing this model-related uncertainty.

## Methods

### Data

Crop model outputs from the GGCMI phase 1 [15] are used, driven by WFDEI.GPCC climate [21], as also used in the ISIMIP2a protocol [17, 22]. Outputs from all GGCMs that have all contributed the necessary scenarios to the GGCMI data archive (n = 10) are used here. These are listed in Table 1, along with their key references. The GGCMs pAPSIM and PEGASUS did not supply data for rice, EPIC-TAMU did not supply data for rice or soybean, ORCHIDEE-crop did not supply data for maize or soybean. Simulations cover the period 1980 to 2009 with one crop-specific growing season per grid cell per year [15].

**Table 1:** GGCMs participating in the study, model type and key references, as well as nutrients considered in crop model simulations (N: nitrogen, P: phosphorus, K: potassium).

Crop model	Model type	Key literature	Nutrients considered
CLM-Crop	Ecosystem Model	Drewniak, Song [23]	N
EPIC-BOKU	Site-based process model (based on EPIC)	EPIC v0810—Williams [24], Izaurralde, Williams [25]	NPK
EPIC-IIASA	Site-based process model (based on EPIC)	EPIC v0810—Williams [24], Izaurralde, Williams [25]	NP
EPIC-TAMU	Site-based process model (based on EPIC)	EPIC v1102—Izaurralde, McGill [26]	NPK
GEPIC	Site-based process model (based on EPIC)	EPIC v0810—Williams [24], Liu, Williams [27]; Folberth, Gaiser [28]	NP
ORCHIDEE-crop	Ecosystem Model	Wu, Vuichard [29]	N
pAPSIM	Site-based process model	APSIM v7.5—Elliott, Kelly [30], Keating, Carberry [31]	NP
pDSSAT	Site-based process model	pDSSAT v1.0—Elliott, Kelly [30]; DSSAT v4.5—Jones, Hoogenboom [32]	NP
PEGASUS	Ecosystem model	v1.1—Deryng, Conway [33], v1.0—Deryng, Sacks [34]	NPK
PEPIC	Site-based process model (based on EPIC)	EPIC v0810—Liu, Yang [35], Liu, Yang [36], Williams [24]	NP

Simulated data is aggregated to global scale time series following simple area-weighted averaging methods [37], using harvested-area data from MIRCA2000 [38]. We compute the different production systems for actual, unlimited, water-limited and nutrient-limited conditions by extracting the corresponding simulation runs from the data archive [15]. These are the *fullharm* setting, in which growing seasons and fertilizer application amounts are harmonized across models [15] and the *harm-suffN* setting (named *harmnon* in [15]), which has the same growing seasons as *fullharm* but assumes no nutrient limitations throughout the simulations. All crop models made simulations under both rainfed and irrigated conditions. The latter assume near-perfect irrigation, leading to very well-watered soils with no limitation of available water inputs, i.e. independent from actual existence of irrigation infrastructures or water supplies. For both settings *fullharm* and *harm-suffN*, fully irrigated and rainfed simulations are available for all crops and grid cells. Crop models have implemented the absence of nutrient limitations on crop yield by either turning nutrient limitations off in their simulations or by supplying large amounts of nutrients during the crop growth simulations. We assume that any differences in these approaches to alleviating nutrient limitation are negligible for the study here.

**Actual simulated yields** are computed from the *fullharm* simulation, combining rainfed and fully irrigated simulations with the area shares on rainfed and irrigated production per crop and grid cell, as specified by the MIRCA2000 data set [38]. **Unlimited water and nutrients yields (uWN)** are taken from the fully irrigated *harm-suffN* data set. **Unlimited nutrient yield (uN)** simulations are taken from the *harm-suffN* simulation data set, using current shares of rainfed and fully irrigated areas per crop and grid cell from MIRCA2000 [38]. **Unlimited water yields (uW)** are computed from the irrigated simulations of the *fullharm* simulation data set. We also compute a data set of **rainfed-limited-nutrients yields (rf)** using rainfed-only *fullharm* simulations to assess the effects of current irrigation systems on yield variability.

We use global yield data from FAOstat [39] for the same period (1980–2009) for a comparison of simulated actual yield variability and observed yield variability. FAOstat data and the globally aggregated simulation data that are shown in Table 2 have been detrended as in Müller, Elliott [18] by subtracting a moving mean of a 5-year window (t-2 to t+2), which is reduced to a 3-year window (t1- to t+1) at both ends of the time series so that the detrended time series is only 2 years shorter (missing t = 1 and t = 30) than the original time series.

**Table 2:** CV of global maize, wheat, rice, and soybean productivity (%) over 28 years (1981–2008) of the 10 individual GGCMs, their ensemble median and FAO statistics [39]. Data are shown for actual, unlimited (uWN), unlimited nutrients (uN) and unlimited water (uW) conditions and have been detrended prior to computing CVs. FAO data is only available for actual conditions. For better readability, the lowest CVs per model (rows) are colored green, highest are colored orange.

Crop	GGCM	actual	uWN	uN	uW
Maize	pDSSAT	3.93	3.31	5.08	2.37
	EPIC-Boku	3.41	2.30	3.53	1.97
	EPIC-IIASA	2.88	2.51	2.97	1.89
	GEPIC	5.10	3.08	4.70	2.88
	pAPSIM	4.13	2.77	4.57	1.79
	PEGASUS	3.82	1.37	2.71	4.16
	CLM-Crop	2.45	2.37	2.44	2.58
	EPIC-TAMU	4.00	2.85	3.90	2.27
	ORCHIDEE-crop	NA	NA	NA	NA
	PEPIC	4.31	1.65	3.70	1.44
	median	3.93	2.51	3.70	2.27
	FAO	4.08	NA	NA	NA
Wheat	pDSSAT	10.13	8.73	9.83	8.93
	EPIC-Boku	3.53	2.25	3.57	2.26
	EPIC-IIASA	8.86	7.93	9.22	8.01
	GEPIC	8.13	7.46	8.31	7.68
	pAPSIM	9.64	8.97	9.86	8.81
	PEGASUS	2.93	2.28	3.46	3.40
	CLM-Crop	3.54	1.48	3.50	1.32
	EPIC-TAMU	8.37	6.84	8.66	7.00
	ORCHIDEE-crop	8.75	6.19	8.82	6.10
	PEPIC	2.94	1.60	3.14	1.44
	median	8.25	6.51	8.48	6.55
	FAO	2.34	NA	NA	NA
Rice	pDSSAT	4.94	5.14	5.70	4.42
	EPIC-Boku	1.16	1.02	1.40	0.86
	EPIC-IIASA	2.58	2.44	2.75	2.32
	GEPIC	2.26	2.76	2.73	2.28
	pAPSIM	NA	NA	NA	NA
	PEGASUS	NA	NA	NA	NA
	CLM-Crop	3.59	2.89	3.69	2.73
	EPIC-TAMU	NA	NA	NA	NA
	ORCHIDEE-crop	1.70	1.58	1.74	1.55
	PEPIC	0.80	1.37	1.15	1.02
	median	2.26	2.44	2.73	2.28
	FAO	1.14	NA	NA	NA
Soybean	pDSSAT	8.09	5.29	8.15	5.36
	EPIC-Boku	5.58	2.79	5.58	2.80
	EPIC-IIASA	4.93	5.86	5.94	4.43
	GEPIC	6.21	4.96	6.06	4.51
	pAPSIM	6.44	5.39	6.42	5.39
	PEGASUS	4.04	3.68	4.04	3.68
	CLM-Crop	11.55	10.30	10.93	11.03
	EPIC-TAMU	NA	NA	NA	NA
	ORCHIDEE-crop	NA	NA	NA	NA
	PEPIC	3.78	2.30	3.62	2.38
	median	5.90	5.12	6.00	4.47
	FAO	3.10	NA	NA	NA

### Metrics

For the analysis, we focus on the relative variability rather than absolute variability to acknowledge that a given variation around a high mean value is less harmful than the same absolute variation around a low mean value. We thus quantify yield variability with the coefficient of variation (CV) in percent (%), i.e. is the standard deviation (*σ*_*x*_) divided by the mean ($\overset{-}{x}$) of the time series x (Eq 1).

$$CV=\frac{\sigma_{x}}{\overset{-}{x}}*100\%$$ (1)

For the analysis of results, we also look at changes in the absolute variability in tonnes dry matter per hectare (t DM ha^-1^), which is represented by the standard deviation of the time series.

As an alternative measure of yield stability, we also compute the distance between the bottom 10% (mean of the lowest yielding 3 years of the 30-year time series, Y_10_) and the time series mean ($\overset{-}{x}$) in t DM ha^-1^, which we refer to as the **yield dent** (Yd, Eq 2). Yd by definition is always positive.

$$Yd=\overset{-}{x}-Y_{10}$$ (2)

## Results

The GGCMs can reproduce observed global yield CV in the *actual* yield simulations for maize but tend to overestimate global yield CV for the other crops, especially for wheat. Models find almost universally that additional water supply (uW) decreases yield CV (Table 2). For additional nutrient supply, the picture is mixed for maize and soybean but a majority of models find that unlimited nutrient supply increases yield variability in wheat (8 of 10) and rice (5 of 7). For wheat (6 of 10) and soybean (5 of 8), most models find that relative yield variability (CV) is reduced most strongly under unlimited water and unlimited nutrient supply, whereas only two models find this for maize and no model finds this for rice. This decrease in relative variability under unlimited water and nutrient supply is mainly driven by an increase in the mean productivity that is larger than the increase of variability, i.e. the absolute variability (*σ*_*x*_) of wheat and soybean yields is found to increase by most models under unlimited water and nutrient supply (Table B in S1 File).

**Fig 1:**
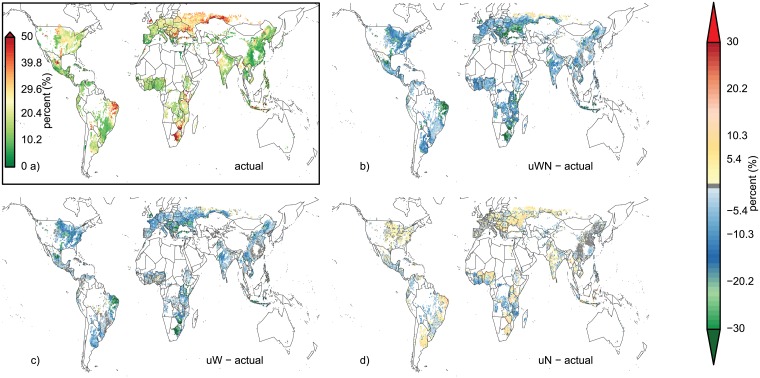
Actual maize yield variability (CV, top right inset) and absolute differences of actual inputs to systems with unlimited water and nutrients (top right), unlimited water (bottom left) and unlimited nutrients (bottom right). Maps show data of the GGCM ensemble median for all grid cells with at least 100ha maize cropland [38] and a minimum yield of 0.5 tDM ha^-1^.

**Fig 2:**
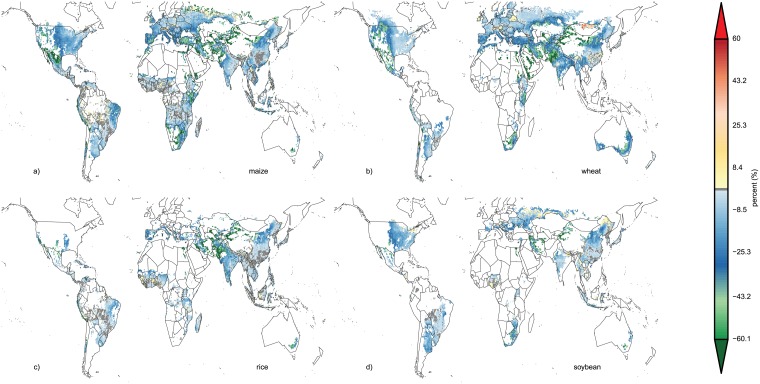
Changes in CV from purely rainfed to fully irrigated systems with current nitrogen (uW-rf). The CV can increase in regions where different growing seasons are specified for irrigated and rainfed systems [15, 38]. Maps show data of the GGCM ensemble median for all grid cells with at least 100ha maize cropland [38] and a minimum yield of 0.5 tDM ha^-1^.

Relative yield variability shows distinct spatial patterns that reflect current variability in weather conditions, the presence of irrigation systems and actual nutrient limitations (Fig 1a for maize, see Figures A-C in S1 File for other crops). The distribution of current irrigation systems (Figure D in S1 File) reflects the patterns where modeled yield CV can be strongly reduced by irrigation which is assumed to be unconstrained by water availability in the simulations, as shown by a comparison of simulated purely rainfed yield CV (rf) and irrigated conditions (Fig 2).

Additional irrigation water would decrease maize yield CV most prominently in south-east Europe, the northeast region of Brazil, southern and eastern Africa, and Indonesia (Fig 2c), a pattern that is even more pronounced under unlimited water and nutrient supply (Fig 2b), whereas unlimited nutrient supply alone would mostly increase maize yield CV in these and most other regions (Fig 2d). The areas where the most pronounced reductions in maize yield CV can be found correspond to regions with high current yield CV (Fig 2a) and low shares of irrigated cropland (Figure D in S1 File).

For wheat, similar effects can be observed (decreasing yield CV under uW and even more pronounced under uWN, but increasing CV under uN), but here the most affected areas are along the Rocky Mountains in the USA and Canada, Australia, the Mediterranean basin and Kazakhstan (Figure A in S1 File).

Rice production systems, which are already mostly irrigated (Figure D in S1 File), show typically a low yield CV under actual conditions and thus show a stronger reduction of yield CV under uN than under uW, although uN can also increase yield CV in some areas, such as in China (Figure B in S1 File).

Soybean, a symbiotic N-fixing crop, shows little response to unlimited nutrient supply. The response of soybean yield CV to unlimited water supply (uW) leads to similar patterns of yield CV (Figure C in S1 File) reductions as for maize and wheat. However, soybean is not cultivated in many dry regions with wheat and/or maize cultivation, as e.g. in Australia or southern Africa, so that there are not as many regions with very strong reductions in yield CV (<-30%).

**Fig 3:**
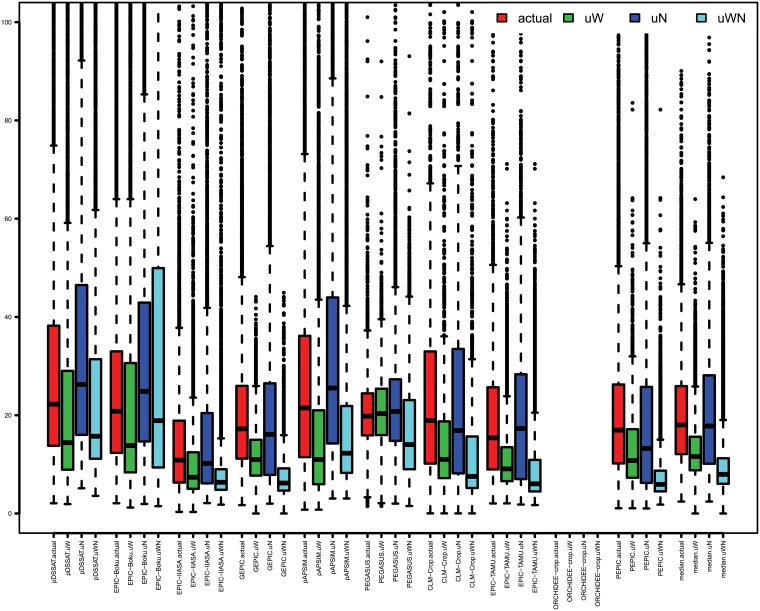
Global distribution of relative (%) temporal yield variability per production system (actual, uW, uN, uWN) and GGCM per grid cell for maize. Colored bars show the interquartile range of yield CVs across all grid cells with at least 100ha maize cropland [38] and a minimum yield of 0.5 tDM ha^-1^. Black lines within the bars show the median, dashed whiskers extend to the maximum value with 1.5 times the interquartile range and values outside this range are classified as outliers and depicted as dots. Yield CV of more than 100% are not shown.

Individual GGCMs show generally the same responses as observed for the GGCM ensemble median but differ in overall simulated variability, but with some model-specific differences (Fig 3). As shown in the GGCM ensemble maps, uWN maize yield CV is reduced compared to actual and uW for most models, but increases compared to uW in pDSSAT, EPIC-Boku, and pAPSIM. Contrary to the other GGCMs, PEGASUS shows a slight increase in maize yield CV under uW compared to actual. Low input systems (for simplicity defined as areas with <60 kg N ha^-1^ year^-1^, Figure E in S1 File) show typically the same patterns as all systems and high input systems (> = 60 kg N ha^-1^ year^-1^, Figure F in S1 File), but low input systems generally show a much stronger maize yield CV than high-input systems and high input systems show a slightly weaker response to uN (Fig 3).

Wheat simulations show the same patterns as do maize simulations, but here EPIC-Boku and EPIC-IIASA show higher yield CV under uWN than under uW and PEGASUS also shows a reduction in wheat yield CV under uW as all other GGCMs do (Figure G in S1 File). For rice, GGCMs typically show little response to additional inputs, but often with uWN showing lowest yield CV. Results from pDSSAT show a much broader yield CV than the other models and also lowest CV under uW (Figure H in S1 File). Soybean simulations also show very little response to additional nutrient inputs (uN), so that actual and uN soybean yield CV are very similar and uW and uWN soybean yield CV are similar to each other as well (Figure I in S1 File).

**Fig 4:**
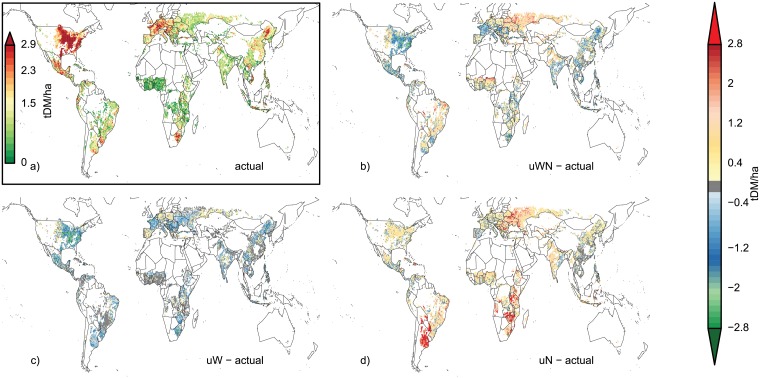
Yield dent (see text) for maize under actual (a) conditions and differences in yield dent for uWN-actual (b), uW-actual (c), and uN-actual (d) for all grid cells with at least 100ha maize cropland [38] and a minimum yield of 0.5 tDM ha^-1^.

The distance between mean yields and the bottom 10% yield (Yd) shows a different response to additional unlimited inputs (Fig 4) than the relative variability (CV). Maize Yd is larger in mostly rainfed areas with high inputs (e.g. USA and western Europe), whereas the maize yield CV is more pronounced in low input regions (Fig 1 and Figure E in S1 File). Also, uW does not generally reduce Yd and uWN shows mixed results. Wheat Yd shows similar response patterns, i.e. mainly increasing Yd under uN and mostly decreasing Yd under uW and mixed response with mainly increasing Yd in low-input regions and decreasing Yd in high-input regions under uWN (Figure J in S1 File). Rice Yd is also less pronounced and shows little response to additional water (uW) with the high irrigation shares in actual rice production (Figure D in S1 File) and thus also mostly increasing Yd under uWN (Figure K in S1 File). Soybean Yd shows little but mixed response to uN and uWN are thus dominated by the decrasing Yd under UW (Figure L in S1 File).

## Discussion and conclusions

Yield variability is driven by a large variety of drivers and mechanisms [1]. Often, weather can explain large parts of overall yield variability, especially in regions with stable management conditions [18, 19]. Additional water inputs often lead to more stable yield productivity across all crops considered here, especially in regions with variable water-deficit years. In the rice producing areas, irrigation shares are already high and generally across all 4 crops, irrigation infrastructure is already implemented in many dry regions (Figure D in S1 File) so that there is low baseline yield CV (e.g. in Spain for maize, Fig 1a and Figure D, panel a in S1 File). In these regions, the effect of the unlimited water scenario (uW) as studied here leads to little reductions in CV (light-blue to grey in Fig 1c). Similarly, additional nutrient supply has little effects on yield CV in high input regions, such as in large parts of eastern China, Europe and the US for maize (Fig 1d). However, these high nutrient-input regions can still display high baseline yield CV, if rainfall is variable and irrigation is not widely applied (e.g. central USA).

Additional nutrient inputs often have two opposing effects. In all nutrient-limited regions, i.e. where crop productivity is not limited by other constraints such as water availability or temperatures, additional nutrient inputs raise mean crop yields and thus decrease relative yield variability (CV). However, bad years (i.e. years with low crop yields) are often defined by adverse weather conditions, so that these do not profit from the additional nutrient inputs. Consequently, absolute yield variability (*σ*_*x*_) and also the yield dent (Yd) increase under additional nutrient inputs, also increasing relative yield variability (CV) compared to actual yields. The overall effect of additional nutrient supply then depends on the dominance of one effect over the other (rising mean yields vs. higher absolute variability). Nutrient input to increase yields has been mainly studied with respect to its spatial variability [40, 41] and limitations by other macronutrients like sulfur (S) and micronutrients like Calcium (Ca) [42] can create “unresponsive soils”. Fertilizer-driven intensification would thus need to be site-specific than assumed in this uniform assessment of eliminating nutrient limitations through fertilizer application [40].

The individual GGCMs largely show similar response patterns, rendering the findings robust. Differences between GGCMs show however that the effects of input-driven intensification are not unequivocal but also depend on other aspects of the production system, such as assumptions on cultivars or soil degradation mechanisms and fertilizer application (e.g. split application vs. single application), which were not harmonized in the simulations here [15]. Also the five EPIC-based GGCMs in the ensemble do not always agree on the effects of additional inputs, which reflects the different assumptions on management systems that strongly affect model performance [43].

Models that are driven with homogenous management assumptions over large areas are bound to overestimate yield variability, because field operations are not conducted in perfect synchrony as in the model setups. Farmers need to stretch the field operations on their land over a longer period of time, as e.g. the machinery or labor force cannot be used across larger areas simultaneously and variation of asynchronously grown fields should cancel out to some extent in the aggregated national statistics. Still, the substantial overestimation of wheat CV by most GGCMs (Table 2) and that of rice and soybean by individual GGCMs warrants further research on model setup [43], data on agricultural management [44] and model implementation and parameters. If homogeneous management assumptions would be the only reason for overestimating yield CV, there should be no substantial difference between the individual crops.

The model setup also assumes near-perfect irrigation on all irrigated land, irrespective of water availability or economic viability, which typically lead to much lower irrigation water use [45] and should thus lead to higher baseline yield variability. Again, as the assumption of near-perfect irrigation should lead to an underestimation of baseline yield CV, reasons for overestimating it need to be better understood in subsequent research.

Fertilizer-driven intensification is likely to increase mean crop yields but also poses a risk to yield stability. This would require additional infrastructure (trade, early warning systems, food storage) on top of the associated nitrogen pollution from the additional inputs, which could be minimized through technological advances [46, 47]. Irrigation-driven intensification on the other hand can increase mean productivity and stabilize crop productivity simultaneously, as it does not lead to yield increases in high-productivity years as with fertilizer-driven intensification but rather in low-productivity years, thus reducing the yield dent. Irrigation can also help to alleviate damages from high-temperature exposure, an effect that is observed for the US [48] and reproduced by GGCMs [49]. But water withdrawals for cropland irrigation are often already unsustainable, violate environmental flow requirements [50] and are projected to be negatively affected by climate change [9]. However, improved irrigation water management [51, 52], in combination with rain water management, may open up new opportunities for sustainable intensification of irrigated crop production [53].

Under given water constraints, large-scale water-driven intensification is less likely than fertilizer-driven intensification, especially as irrigation water supply is also subject to climate change [9]. Our analysis shows that fertilizer-driven intensification is generally possible, but would imply increasing inter-annual variability of crop yields. In this case, trade and storage networks, infrastructure and policies will have to prepare to buffer against low harvest events in individual regions in order to avoid food price volatility [4]. This will become even more important under climate change, which may increase the frequency and duration of extreme weather events [54]. Selection of adequate crop varieties may also help to reduce variability in crop production [12].

## Supporting information

S1 File The supplementary information (SI) is provided in one single PDF file. **Table A**. Global time series correlation coefficients of the actual simulations and the reported national time series (FAO statistics [1]). Data taken from Müller, Elliott [2]. Some models did not supply data for all crops, which is indicated with NA. Some correlation coefficients are not statistically significant, which is indicated with ‘(ns)’. **Table B**. Standard deviation of global maize, wheat, rice, and soybean productivity (t DM/ha) over 30 years (1980–2009) of 9 individual GGCMs, their ensemble median and FAO statistics [1]. Data are shown for actual, unlimited (uWN), unlimited nutrients (uN) and unlimited water (uW) conditions. FAO data is only available for actual conditions. Lowest standard deviations per model are colored green, highest are colored orange. **Figure A**. same as Fig 1 of the main text, but for wheat. **Figure B**. same as Fig 1 of the main text, but for rice. **Figure C**. same as Fig 1 of the main text, but for soybean. **Figure D**. shares of irrigated crop land in total cropland per crop (maize, wheat, rice, soybean) in percent as specified by the MIRCA2000 data set [3]. **Figure E**. as Fig 3 of the main text but for low input systems (<60 kgN ha-1 year-1) only. **Figure F**. same as Fig 3 of the main text but for high input systems (> = 60 kgN ha-1 year-1) only. **Figure G**. Same as Fig 3 of the main text, but for wheat. **Figure H**. same as Fig 3 of the main text, but for rice. **Figure I**. Same as Fig 3 of the main text, but for soybean. **Figure J**. Same as Fig 4 of the main text, but for wheat. **Figure K**. Same as Fig 4 of the main text, but for rice. **Figure L**. Same as Fig 4 of the main text, but for soybean.
(PDF)
