# Supplementary material for: Global patterns of crop yield stability under additional nutrient and water inputs
Source: PLoS One. 2018 Jun 27;13(6):e0198748. doi: 10.1371/journal.pone.0198748 (PMC6021068; doi:10.1371/journal.pone.0198748)
Supplement: S1 File — Table A. Global time series correlation coefficients of the actual simulations and the reported national time series (FAO statistics [1]). Data taken from Müller, Elliott [2]. Some models did not supply data for all crops, which is indicated with NA. Some correlation coefficients are not statistically significant, which is indicated with ‘(ns)’. Table B. Standard deviation of global maize, wheat, rice, and soybean productivity (t DM/ha) over 30 years (1980–2009) of 9 individual GGCMs, their ensemble median and FAO statistics [1]. Data are shown for actual, unlimited (uWN), unlimited nutrients (uN) and unlimited water (uW) conditions. FAO data is only available for actual conditions. Lowest standard deviations per model are colored green, highest are colored orange. Figure A. same as Fig 1 of the main text, but for wheat. Figure B. same as Fig 1 of the main text, but for rice. Figure C. same as Fig 1 of the main text, but for soybean. Figure D. shares of irrigated crop land in total cropland per crop (maize, wheat, rice, soybean) in percent as specified by the MIRCA2000 data set [3]. Figure E. as Fig 3 of the main text but for low input systems (<60 kgN ha-1 year-1) only. Figure F. same as Fig 3 of the main text but for high input systems (> = 60 kgN ha-1 year-1) only. Figure G. Same as Fig 3 of the main text, but for wheat. Figure H. same as Fig 3 of the main text, but for rice. Figure I. Same as Fig 3 of the main text, but for soybean. Figure J. Same as Fig 4 of the main text, but for wheat. Figure K. Same as Fig 4 of the main text, but for rice. Figure L. Same as Fig 4 of the main text, but for soybean. (PDF) [file pone.0198748.s001.pdf]

Supplementary material

Global patterns of crop yield stability under additional nutrient and water inputs

Christoph Müller[1,*], Joshua Elliott[2,3], Thomas A. M. Pugh[4,5], Alex C. Ruane[3,6], Philippe Ciais[7], Juraj Balkovic[8,9], Delphine Deryng[3,10], Christian Folberth[8], R. Cesar Izaurralde[11,12], Curtis D. Jones[11], Nikolay Khabarov[8], Peter Lawrence[13], Wenfeng Liu[7,14], Ashwan D. Reddy[11], Erwin Schmid[15], Xuhui Wang[7,16]

[1] *Potsdam Institute for Climate Impact Research, Potsdam, Germany*
[2] *University of Chicago and ANL Computation Institute, Chicago, USA*
[3] *Columbia University Center for Climate Systems Research, New York, USA*
[4] *School of Geography, Earth & Environmental Science and Birmingham Institute of Forest Research, University of Birmingham, Birmingham, United Kingdom*
[5] *IMK-IFU, Karlsruhe Institute of Technology, Garmisch-Partenkirchen, Germany.*
[6] *National Aeronautics and Space Administration Goddard Institute for Space Studies, New York, USA*
[7] *Laboratoire des Sciences du Climat et de l'Environnement. Gif-sur-Yvette, France*
[8] *Ecosystem Services and Management Program, International Institute for Applied Systems Analysis, Laxenburg, Austria*
[9] *Department of Soil Science, Faculty of Natural Sciences, Comenius University in Bratislava, Bratislava, Slovak Republic*
[10] *Climate Analytics, Berlin, Germany*
[11] *University of Maryland, Department of Geographical Sciences, College Park, USA*
[12] *Texas A&M University, Texas AgriLife Research and Extension, Temple, USA*
[13] *National Center for Atmospheric Research, Earth System Laboratory, Boulder, USA*
[14] *Eawag, Swiss Federal Institute of Aquatic Science and Technology, Duebendorf, Switzerland*
[15] *Institute for Sustainable Economic Development, University of Natural Resources and Life Sciences, Vienna, Austria*
[16] *Sino-French Institute of Earth System Sciences, Peking University, Beijing, China*

* Corresponding author

Christoph.Mueller@pik-potsdam.de

Table A: Global time series correlation coefficients of the actual simulations and the reported national time series (FAO statistics [1]). Data taken from Müller, Elliott [2]. Some models did not supply data for all crops, which is indicated with NA. Some correlation coefficients are not statistically significant, which is indicated with '(ns)'.

GGCM	Maize	Wheat	Rice	Soybean
pDSSAT	0.843	0.531	0.288 (ns)	0.544
EPIC-Boku	0.663	0.385	0.351	0.603
EPIC-IIASA	0.700	0.597	0.187 (ns)	0.278 (ns)
GEPIC	0.749	0.371	0.119	0.565
pAPSIM	0.774	0.346	NA	0.615
PEGASUS	0.224 (ns)	0.299 (ns)	NA	0.254 (ns)
CLM-Crop	0.295 (ns)	0.299 (ns)	0.161 (ns)	0.326 (ns)
EPIC-TAMU	0.667	0.545	NA	NA
ORCHIDEE-crop	0.548	0.395	0.256 (ns)	0.266 (ns)
PEPIC	0.687	0.397	0.373	0.389

Table B: Standard deviation of global maize, wheat, rice, and soybean productivity (t DM/ha) over 30 years (1980-2009) of 9 individual GGCMs, their ensemble median and FAO statistics [1]. Data are shown for actual, unlimited (uWN), unlimited nutrients (uN) and unlimited water (uW) conditions. FAO data is only available for actual conditions. Lowest standard deviations per model are colored green, highest are colored orange.

Crop	GGCM	actual	uWN	uN	uW
maize	pDSSAT	0.272	0.345	0.440	0.186
	EPIC-Boku	0.207	0.170	0.221	0.140
	EPIC-IIASA	0.144	0.182	0.191	0.100
	GEPIC	0.227	0.224	0.273	0.144
	pAPSIM	0.195	0.214	0.286	0.096
	PEGASUS	0.106	0.174	0.267	0.128
	CLM-Crop	0.162	0.200	0.169	0.206
	EPIC-TAMU	0.191	0.232	0.238	0.135
	ORCHIDEE-crop				
	PEPIC	0.200	0.124	0.226	0.076
	median	0.195	0.200	0.238	0.135
	FAO	0.165			
wheat	pDSSAT	0.500	0.745	0.628	0.580
	EPIC-Boku	0.108	0.101	0.116	0.094
	EPIC-IIASA	0.230	0.374	0.316	0.238
	GEPIC	0.219	0.373	0.313	0.232
	pAPSIM	0.303	0.633	0.488	0.320
	PEGASUS	0.109	0.266	0.252	0.172
	CLM-Crop	0.100	0.054	0.101	0.047
	EPIC-TAMU	0.180	0.319	0.289	0.179
	ORCHIDEE-crop	0.262	0.324	0.285	0.285
	PEPIC	0.074	0.074	0.117	0.040
	median	0.180	0.319	0.289	0.179
	FAO	0.059			
rice	pDSSAT	0.169	0.207	0.226	0.156
	EPIC-Boku	0.029	0.028	0.036	0.022
	EPIC-IIASA	0.073	0.090	0.100	0.067
	GEPIC	0.072	0.124	0.119	0.074
	pAPSIM				
	PEGASUS				
	CLM-Crop	0.079	0.080	0.087	0.071
	EPIC-TAMU				
	ORCHIDEE-crop	0.102	0.101	0.107	0.096
	PEPIC	0.030	0.066	0.054	0.039

	median	0.072	0.085	0.093	0.069
	FAO	0.041			
soybean	pDSSAT	0.230	0.222	0.237	0.222
	EPIC-Boku	0.140	0.079	0.140	0.079
	EPIC-IIASA	0.110	0.172	0.163	0.101
	GEPIC	0.151	0.164	0.170	0.118
	pAPSIM	0.197	0.215	0.198	0.215
	PEGASUS	0.077	0.080	0.077	0.080
	CLM-Crop	0.233	0.233	0.233	0.239
	EPIC-TAMU				
	ORCHIDEE-crop				
	PEPIC	0.091	0.063	0.091	0.061
	median	0.146	0.168	0.167	0.109
	FAO	0.063			

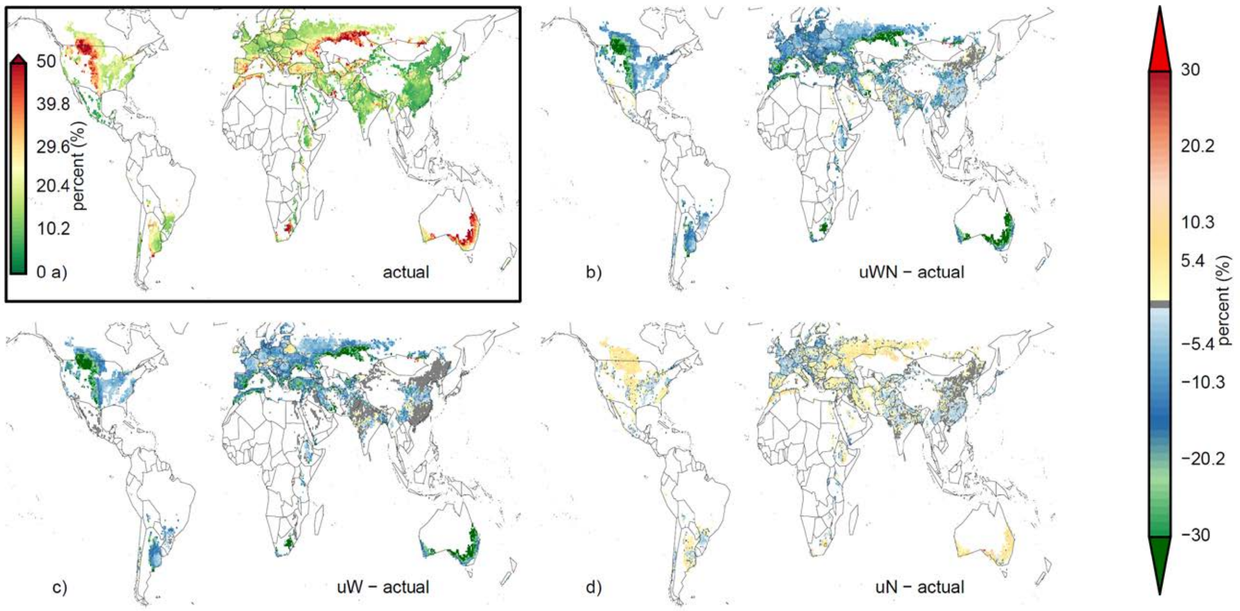

Figure A: same as Fig 1 of the main text, but for wheat

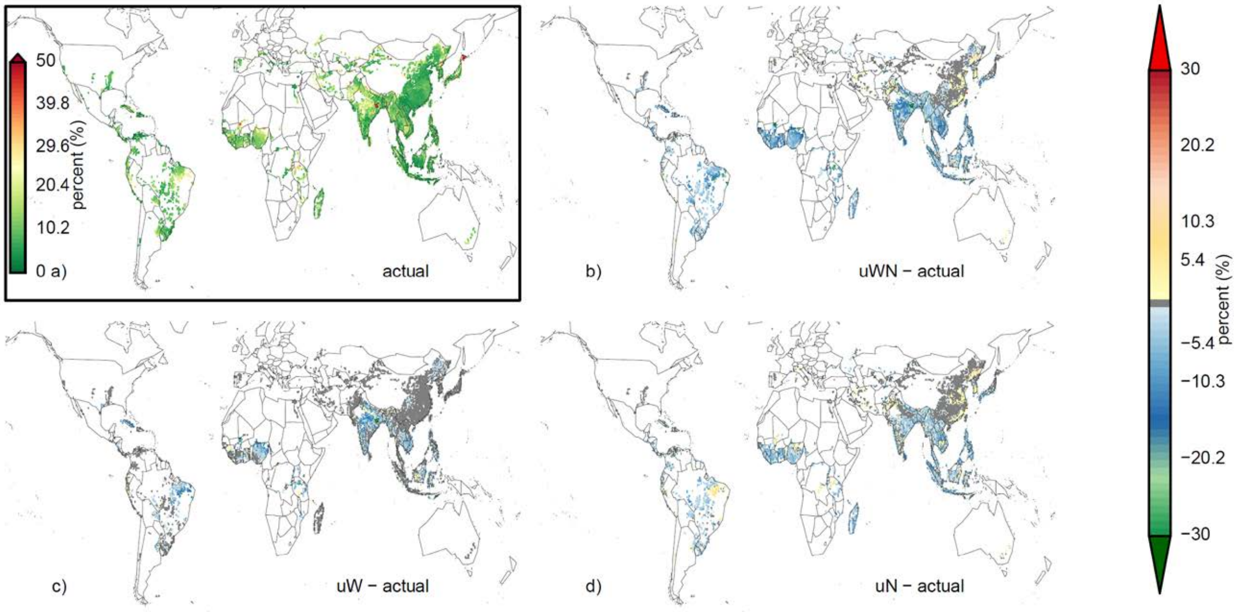

Figure B: same as Fig 1 of the main text, but for rice

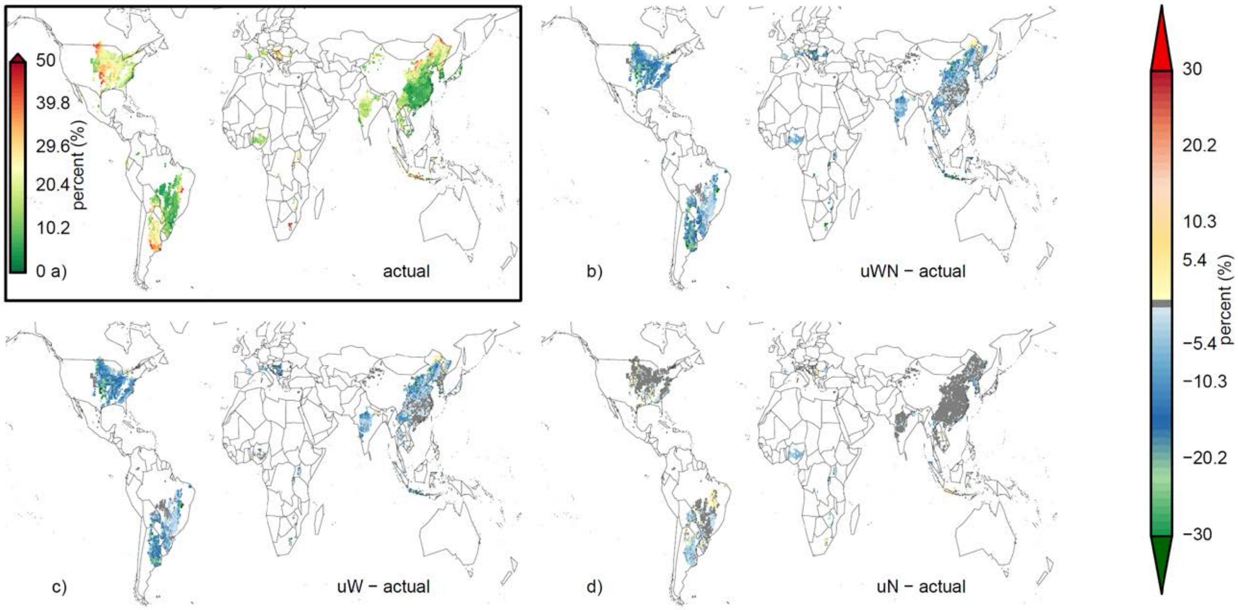

Figure C: same as Fig 1 of the main text, but for soybean

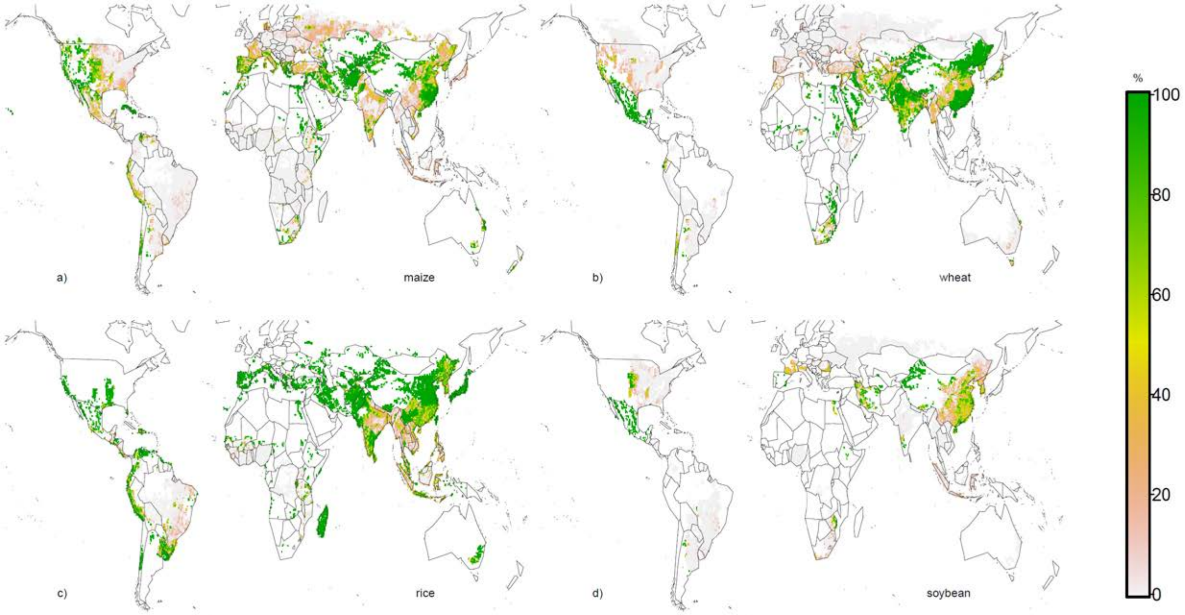

Figure D: shares of irrigated crop land in total cropland per crop (maize, wheat, rice, soybean) in percent as specified by the MIRCA2000 data set [3].

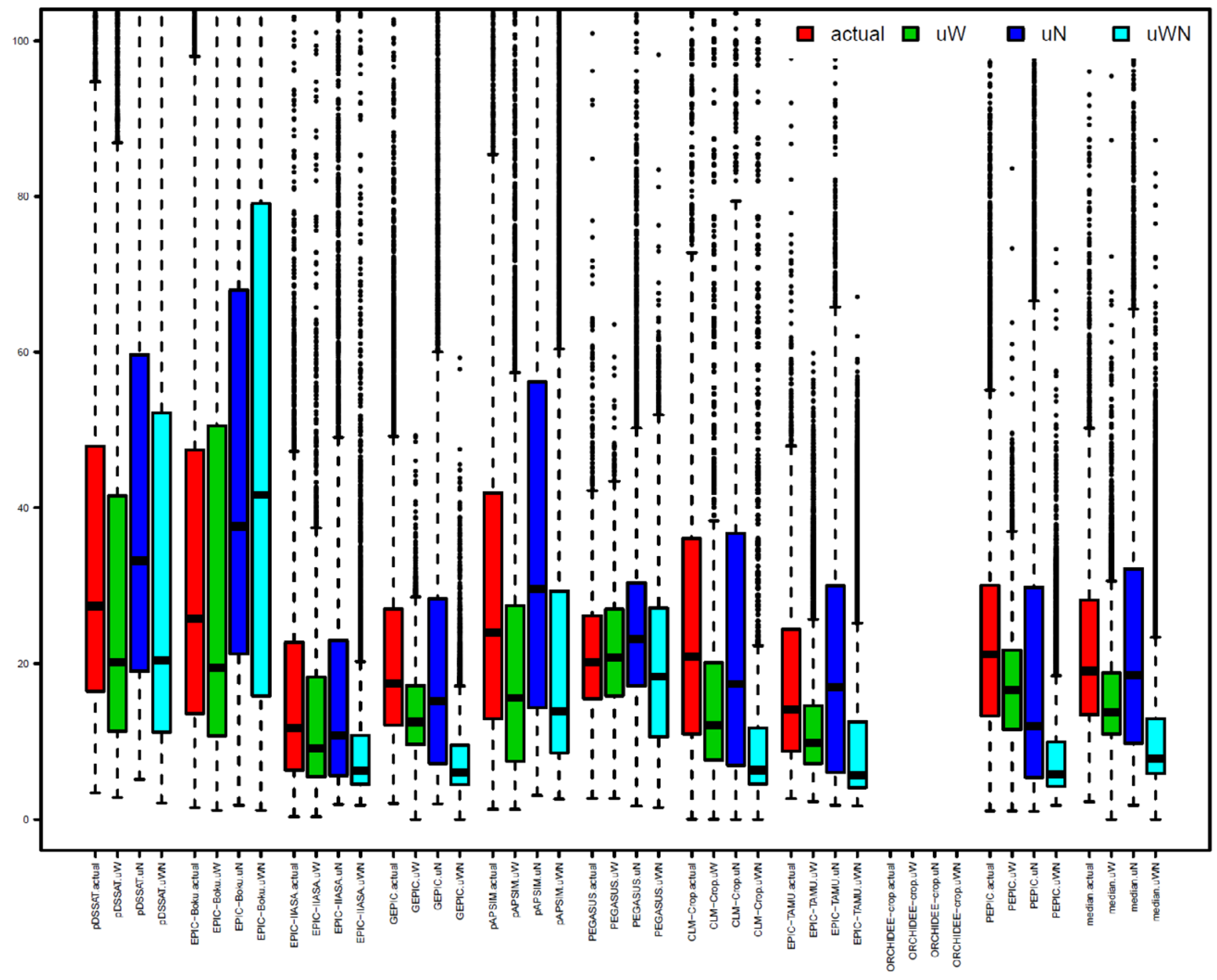

Figure E: as Fig 3 of the main text but for low input systems (<60 kgN ha^{-1} $year^{-1}$) only.

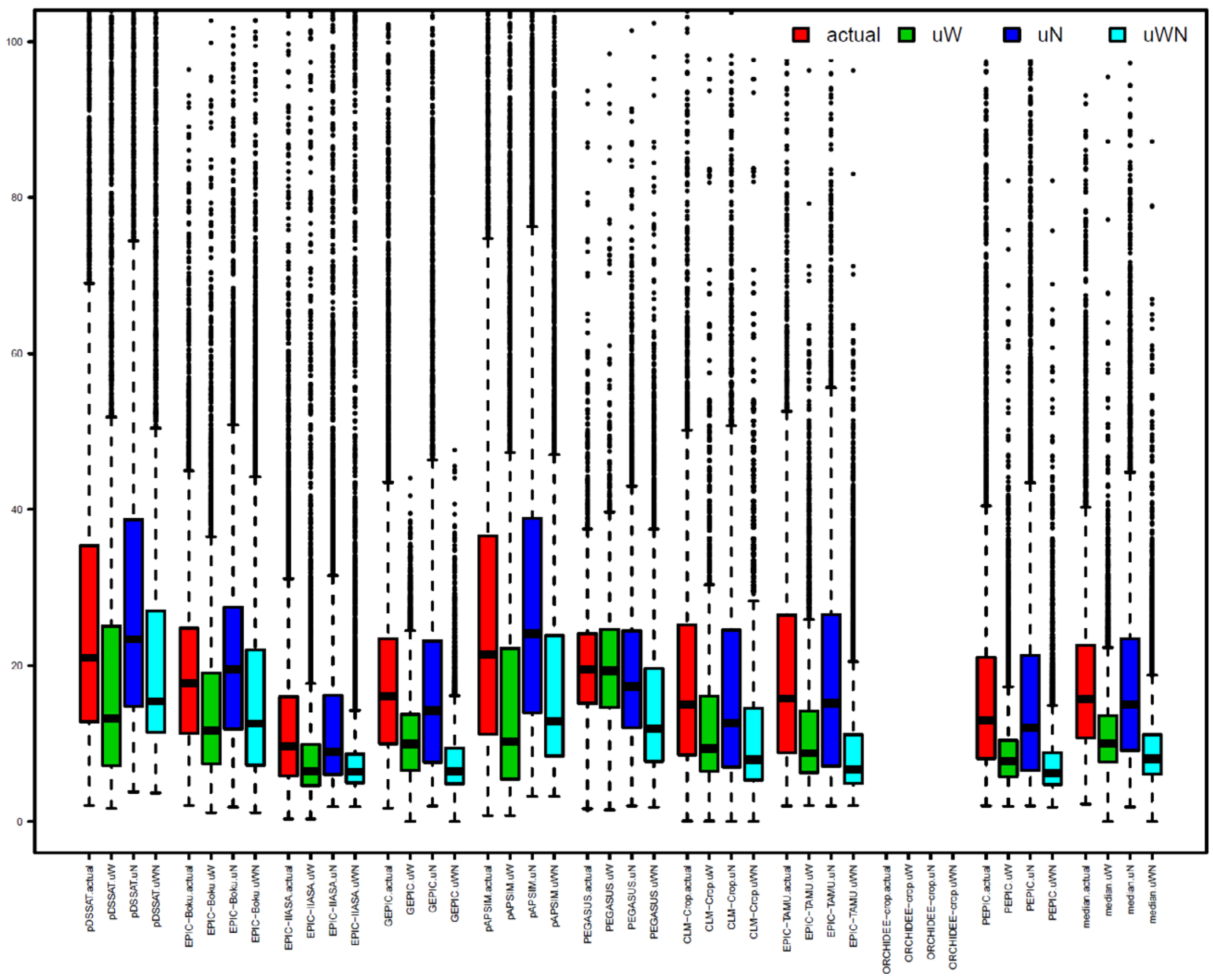

Figure F: same as Fig 3 of the main text but for high input systems (>=60 kgN ha^{-1} year^{-1}) only.

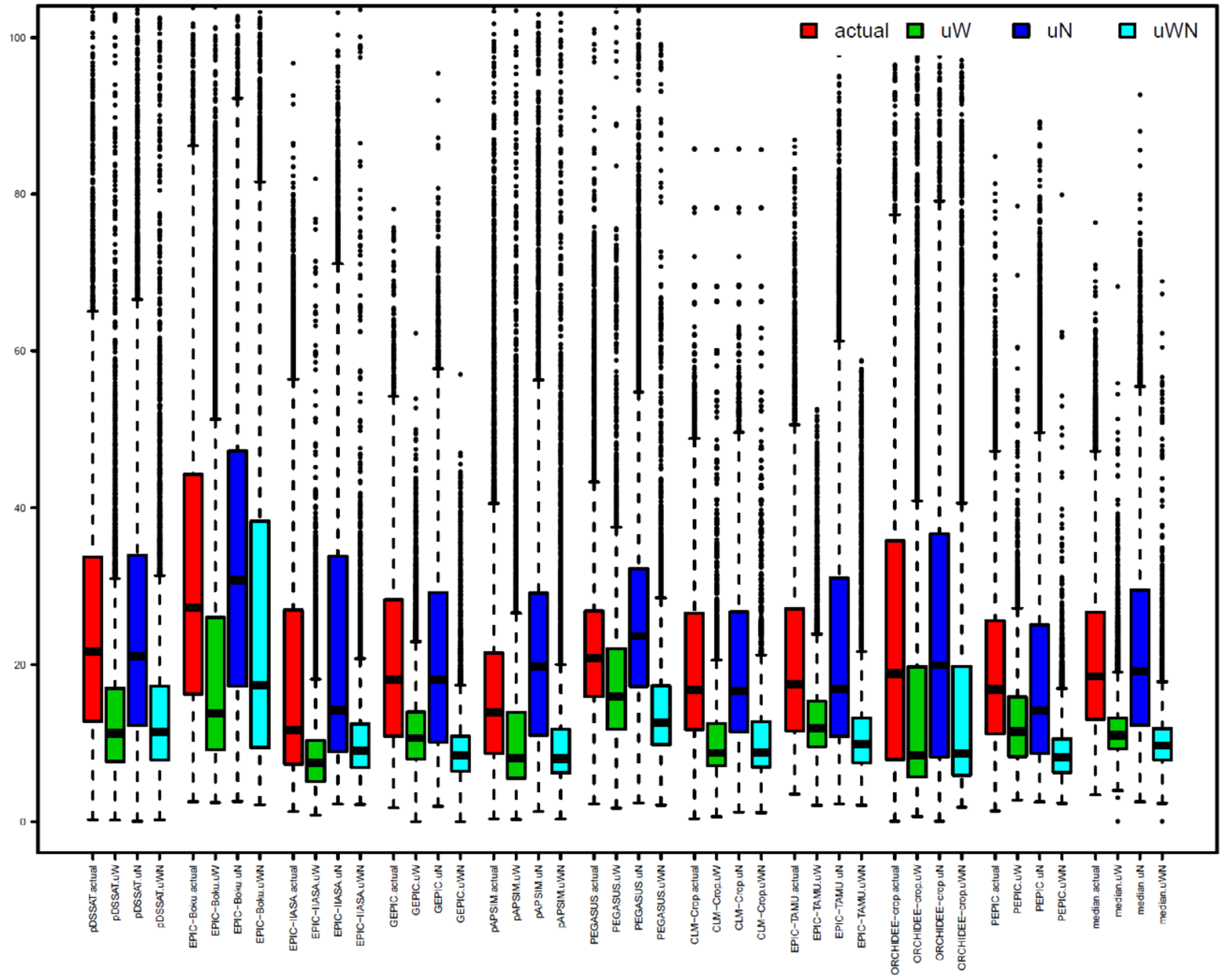

Figure G: Same as Fig 3 of the main text, but for wheat.

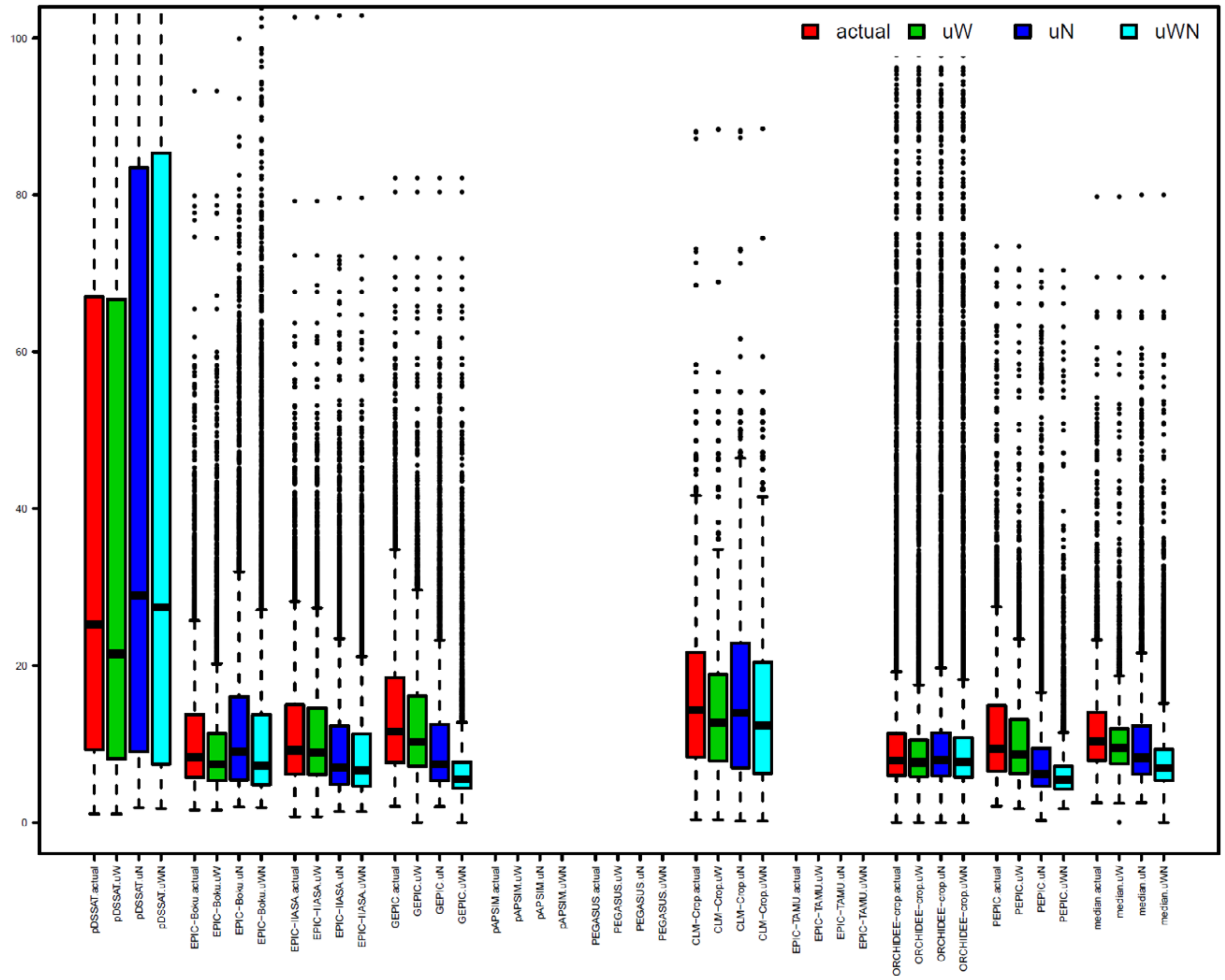

Figure H: same as Fig 3 of the main text, but for rice.

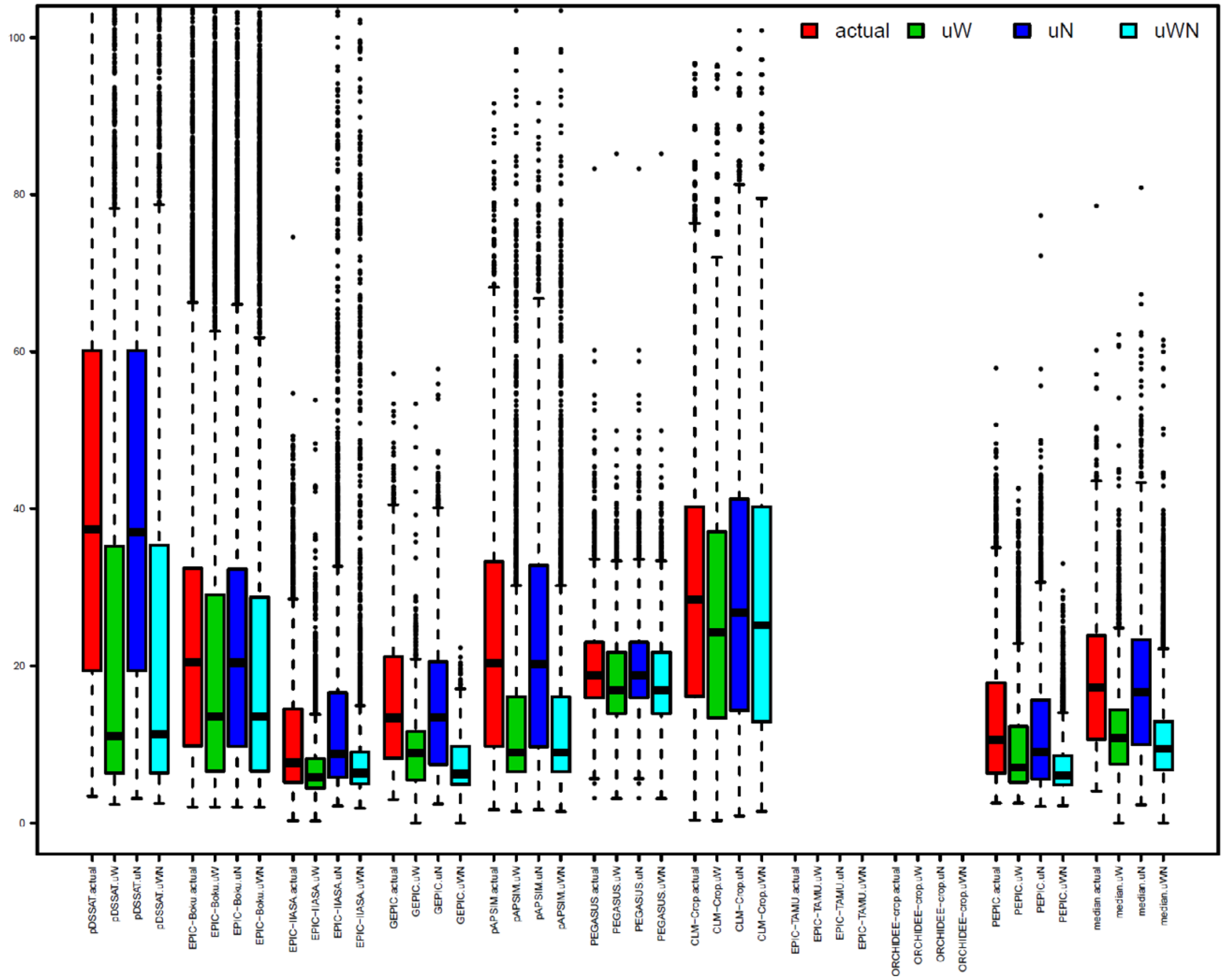

Figure I: Same as Fig 3 of the main text, but for soybean.

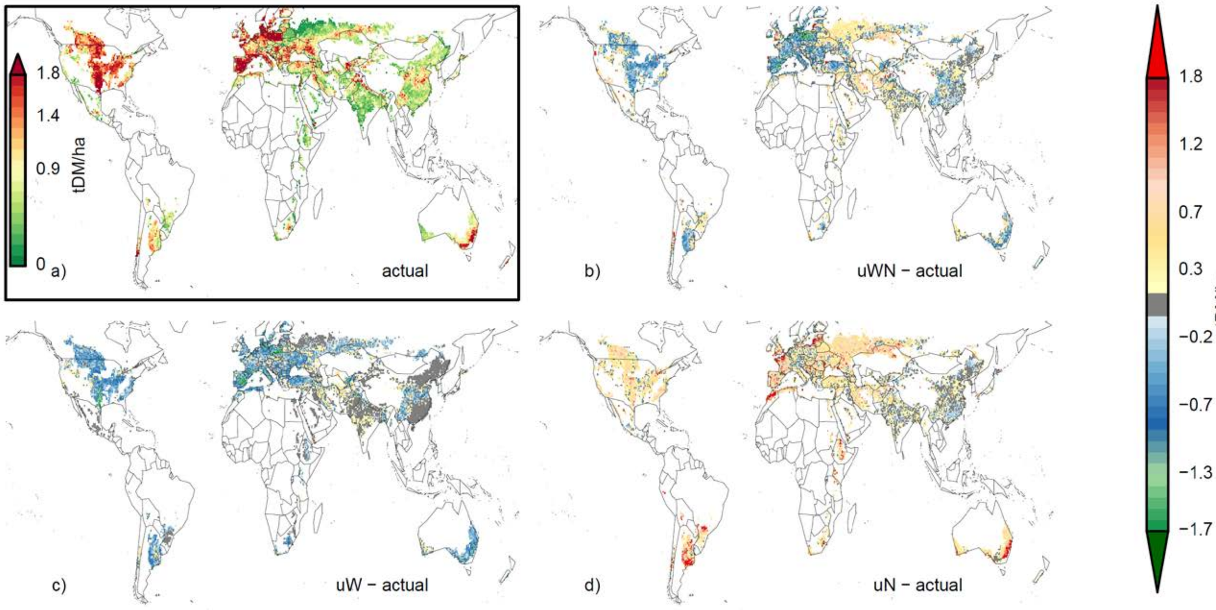

Figure J: Same as Fig 4 of the main text, but for wheat.

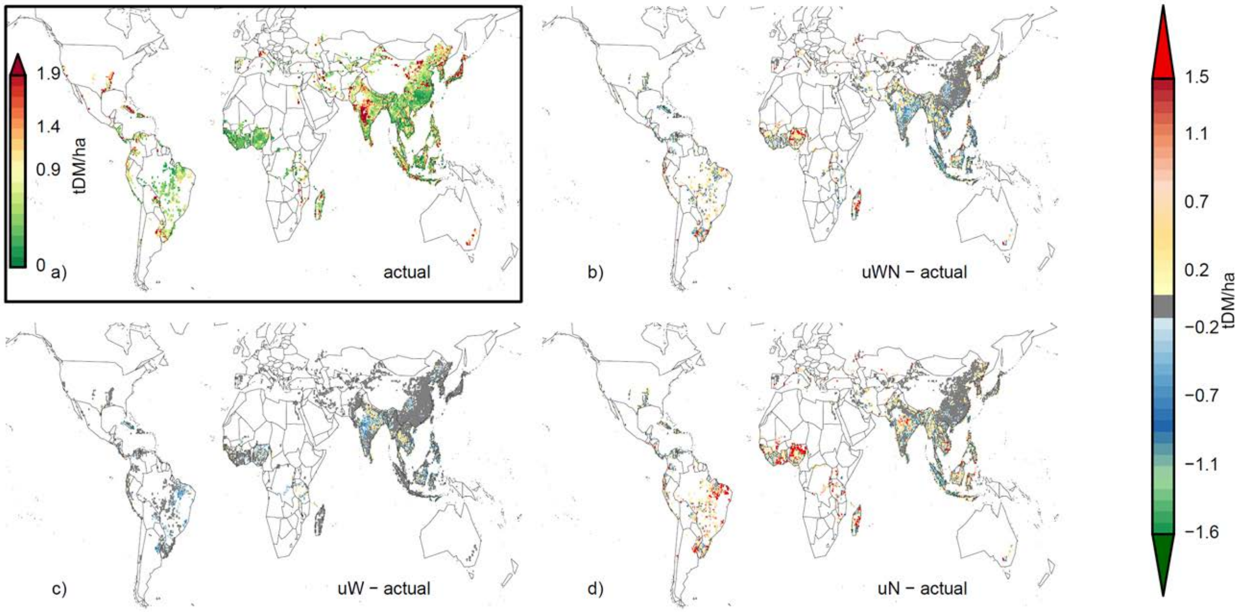

Figure K: Same as Fig 4 of the main text, but for rice

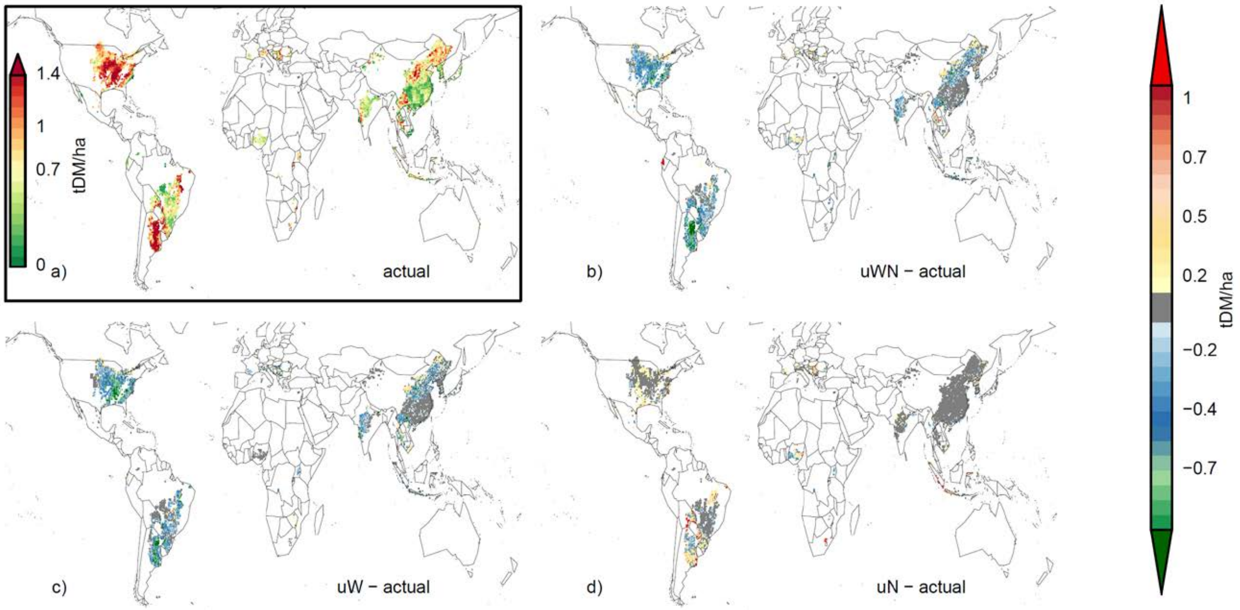

Figure L: Same as Fig 4 of the main text, but for soybean.

References

1. FAOstat data. http://faostat3.fao.org/home/E [accessed: May 2014]. 2014.
2. Müller C, Elliott J, Chryssanthacopoulos J, Arneth A, Balkovic J, Ciais P, et al. Global gridded crop model evaluation: benchmarking, skills, deficiencies and implications. Geosci Model Dev. 2017;10(4):1403-22. doi: 10.5194/gmd-10-1403-2017.
3. Portmann FT, Siebert S, Döll P. MIRCA2000-Global monthly irrigated and rainfed crop areas around the year 2000: A new high-resolution data set for agricultural and hydrological modeling. Global Biogeochemical Cycles. 2010;24:Gb1011. doi: 10.1029/2008gb003435. PubMed PMID: ISI:000275495000001.
